# Potential of Egg White Protein-Based Films for Maintaining the Quality of Fresh-Peeled Garlic

**DOI:** 10.3390/foods15162828

**Published:** 2026-08-14

**Authors:** Víctor Baquero-Aznar, Sara Vega-Diez, Bianca Souza da Costa, María Luisa Salvador, Jaime González-Buesa

**Affiliations:** 1 Departamento de Ciencia Vegetal, Instituto Agroalimentario de Aragón—IA2, Centro de Investigación y Tecnología Agroalimentaria de Aragón (CITA), Av. Montañana 930, 50059 Zaragoza, Spain; vbaquero@cita-aragon.es (V.B.-A.); svega@cita-aragon.es (S.V.-D.); bdesouza@cita-aragon.es (B.S.d.C.); 2 Grupo de Investigación en Alimentos de Origen Vegetal, Instituto Agroalimentario de Aragón—IA2, Universidad de Zaragoza, Miguel Servet 177, 50013 Zaragoza, Spain; mlsalva@unizar.es

**Keywords:** beeswax, multilayer films, edible films, egg white protein, garlic, microperforated films, modified atmosphere packaging

## Abstract

Fresh-peeled garlic cloves are a very convenient ready-to-cook product; however, their high perishability requires packaging systems that maintain the quality of the garlic while addressing the need for more sustainable materials. This study evaluated the quality evolution of peeled garlic cloves during refrigerated storage (5 °C) in microperforated modified atmosphere packaging (MAP) systems consisting of trays sealed with egg white protein (EWP)-based films, either uncoated (EWP-U) or coated with beeswax (EWP-BW). Their performance was compared with commercial polylactic acid (PLA) and oriented polypropylene (OPP) films. The EWP-based packages generated an internal atmosphere of approximately 7% O_2_ and 15% CO_2_, under which peeled garlic cloves showed delayed fungal decay, reduced yeast and mold growth, and mitigated surface discoloration compared with other packaging systems, whose atmospheres remained closer to air. However, weight loss was promoted in the garlic cloves packaged with EWP-U films. The hydrophobic coating applied in EWP-BW films improved the water vapor barrier properties compared with EWP films, thus reducing the weight loss observed in the garlic cloves, but increasing fungal decay. These results suggest that an optimized packaging system should combine the lower water vapor transmission rate provided by EWP-BW films with the internal gas composition achieved in EWP-U packages. Accordingly, EWP-BW films represent a promising bio-based alternative for preserving the quality of peeled garlic cloves, provided that the effective O_2_ and CO_2_ transmission rates through the package are appropriately adjusted to generate a more favorable modified atmosphere.

## 1. Introduction

Garlic (*Allium sativum*) is an edible plant member of the onion family (Alliaceae) known for its health-promoting function, demonstrating antioxidant, anti-inflammatory, antibacterial, antifungal, and immune-boosting properties [1]. It is considered an important culinary ingredient worldwide due to its unique strong aroma and pungent flavor, which are associated with sulfur-containing compounds such as allicin and alliin [2].

Peeled garlic cloves have emerged as a ready-to-cook product in retail and food-service markets due to their convenience, as they save significant preparation time, reduce waste, and eliminate the need to remove outer and inner peels and handle sticky cloves. The handling, processing, and removal of the different protective layers of the garlic clove significantly reduce the product’s shelf life; garlic bulbs can be kept for more than 9 months at low temperatures, while the expected shelf life of peeled garlic is around 2–3 weeks [3]. To overcome this limitation, modified atmosphere packaging (MAP) combined with refrigeration has been applied to peeled garlic cloves. This method has proven to be an effective strategy to extend the shelf life by slowing down discoloration, browning, the loss of pungent flavor, and microbial activity [1,4,5,6,7]. The recommended gas compositions normally combine low O_2_ concentrations (3%) and moderate CO_2_ levels (15%) [8], although garlic has demonstrated a good tolerance of higher CO_2_ concentrations over short storage periods [3].

Unfortunately, MAP normally involves the use of petroleum-based packages, and there is a global concern over plastic pollution and the environmental burden of these packaging materials [9]. Over the years, a wide range of bio-based materials have been investigated as sustainable alternatives to petroleum-based materials for the preservation of different horticultural products. Among these materials, protein-based polymers stand out due to their intrinsic film-forming ability [10,11], excellent oxygen barrier performance [11], and capacity to incorporate functional additives [12]. In particular, egg white protein (EWP) is abundant, is nutritionally valuable, and exhibits favorable functional and processing characteristics [13,14]. As with other protein-derived films, its broader application is constrained by its inherent drawbacks, such as poor moisture barrier properties resulting from the protein’s hydrophilic amino acid composition, mechanical brittleness, and high sensitivity to humidity and temperature [15].

To overcome these limitations, a range of strategies have been explored. These approaches include the physical [16] and chemical modification of the EWP’s molecular structure [9,17], enzymatic cross-linking [18], and blending with polysaccharides [18,19,20] or other proteins, such as soy [21]. The incorporation of nanoparticles has also been shown to significantly improve the mechanical, barrier, and antibacterial properties of EWP-based films [22,23].

Beyond these strategies, multilayer film structures, where each layer plays a specific (technical) function, have emerged as an option to enhance the performance of protein-based materials. Lipids and waxes, unlike proteins, provide strong resistance to moisture; however, they cannot form self-supporting monolayers due to their poor mechanical strength. To address this limitation, bilayer systems combining proteins with natural waxes have been proposed, thereby integrating the complementary advantages of both components [24]. Beeswax (BW), a natural wax produced by honey bees, is a highly hydrophobic material representing a suitable option in this context. Incorporating BW into protein-based films offers a promising strategy to enhance their water resistance [24,25]. Bilayer or multilayer structures can be obtained either by laminating BW over polymeric films using a layer-by-layer coating technique, or by dispersing BW uniformly within a polymeric emulsion, followed by lipid migration to the surface to form pseudo-bilayer systems [25,26]. Most studies on bilayer or multilayer systems incorporating BW have been conducted with films derived from polylactic acid [27], polysaccharides such as agar/maltodextrin [25], or even paper and paperboard substrates [28]. In contrast, limited attention has been given to the development of protein-based multilayer films using BW, despite their potential to address the limitations of protein monolayers.

EWP-based films are characterized in terms of their microstructure, thermal resistance, and mechanical, barrier, and optical properties to determine the extent to which different alternatives enhance their functional performance. However, only a limited number of studies have evaluated their influence on the quality and shelf life of packaged foods by using edible coatings or wraps [29,30,31,32,33,34]. Moreover, most studies on protein-based films intended for modified-atmosphere packaging have relied on films produced by solution casting. By contrast, compression molding treats plasticized proteins as thermoprocessable biopolymeric materials and is therefore more closely aligned with conventional industrial plastic-conversion processes, as film formation is achieved through the combined application of heat and pressure rather than solvent evaporation. Nevertheless, the development of real packages from EWP to preserve food has scarcely been addressed, with some exceptions such as oil packaging [35] and cherry tomato packaging [36]. To date, the potential of compression-molded EWP multilayer films for MAP applications remains unexplored, and this study sought to fill this gap in their applicability.

This study aimed to evaluate the potential of EWP-based films for the MAP of minimally processed products such as peeled garlic cloves. The main objectives of this work were (i) to develop and characterize EWP-based films produced by compression molding, with and without a beeswax coating, to assess whether the multilayer material improves the water resistance of EWP films, and (ii) to analyze the quality evolution of peeled garlic stored in packages made from these films, compared with commercial films such as OPP and PLA. The films were evaluated for oxygen and water vapor permeability at different relative humidities (RHs) and for their optical properties. The evolution of the gas composition and the main quality parameters of the garlic cloves were monitored throughout storage, including weight loss, the rot incidence, the color, the firmness, the phenolic content, the pungency of the flavor, and microbiological growth.

## 2. Materials and Methods

### 2.1. Materials

EWP powder was supplied by Eurovo (Valladolid, Spain). Food-grade glycerin was purchased from Barcelonesa Global Chemical Solutions (Cornellà de Llobregat, Spain). Beeswax was sourced from Sigma-Aldrich (St. Louis, MO, USA). The oriented polypropylene (OPP) film was provided by Envaflex (Utebo, Spain), and the polylactic acid (PLA) film was supplied by Sidaplax (Ghent, Belgium). Peeled garlic cloves (cv. ajo morado español) were obtained from Ajos Torres (Alpartir, Spain) and stored at 4.7 ± 0.2 °C and a 66.5 ± 1.7% relative humidity (RH) for no longer than 24 h prior to packaging. The Folin–Ciocalteu reagent and gallic acid were purchased from Sigma-Aldrich (Steinheim, Germany). All other excipients and chemicals used in the analysis were of analytical grade.

### 2.2. Preparation of EWP-Based Films

The film-forming solution (FFS) was prepared by dispersing EWP powder, glycerin, and water at a ratio of 1:0.5:0.5 (*w*/*w*), using an orbital mixer (KitchenAid, Benton Harbor, MI, USA) for 30 min at room temperature under vacuum conditions. The FFS was introduced in 20 mL syringes to avoid oxidation or dehydration and stored at 5 °C for 24 h to stabilize. Subsequently, 10 g of the FFS was uniformly distributed in small amounts on aluminum foil using a 2.5 cm squared grid as a guide, and another piece of aluminum foil was placed over the sample. The sample together with the aluminum sheets was placed in an LP-S-50 laboratory hot-plate press (Labtech Engineering Co., Samutprakarn, Thailand) and processed through compression molding. Processing comprised two consecutive cycles: heating at 130 °C under 150 bars for 5 min, followed by cooling at 20 °C under 150 bars for 5 min, as described previously [9,22,36]. To apply the beeswax layer, the EWP films were immersed in molten beeswax at 70 °C for 5 s, and the excess beeswax was drained off by gravity. The coating solidified rapidly at room temperature, yielding a BW coating on both sides of the EWP film. The coated films were visually inspected and showed complete and visually homogeneous surface coverage, with no detectable visibly uncoated areas. The uniformity of the resulting films was also evaluated by measuring the thickness at different locations on the samples and inspecting them under an optical microscope for smaller defects. Samples with defects or a non-uniform thickness were discarded. Uncoated and beeswax-coated films were conditioned at 23 °C and 55% RH for at least 2 days before testing.

### 2.3. Film Characterization

#### 2.3.1. Oxygen Permeability (*OP*) and Water Vapor Permeability (*WVP*)

The oxygen transmission rate (*OTR*) was determined on an Ox-Tran^®^ 2/22 (MOCON Inc., Minneapolis, MN, USA) in accordance with the standard ASTM D3985-17 [37], and the water vapor transmission rate (*WVTR*) was investigated using a Permatran-W^®^ 3/34 (MOCON Inc., Minneapolis, MN, USA) following the standard ASTM F1249-20 [38]. To evaluate the moisture sensitivity of the films, the *OTR* was measured at 23 °C and RHs of 0, 30, 50, 70, and 90%, while the *WVTR* was measured at 23 °C and RHs of 30, 50, 70, and 90%. For each film material, six independently prepared specimens were tested. The film thickness (e) was determined as the mean of the measurements taken at five positions using a 547-401 digital thickness gauge (Mitutoyo Corp., Kanagawa, Japan). The oxygen and water vapor permeabilities (*OP* and *WVP*, respectively) were calculated from the corresponding transmission rates (*OTR* and *WVTR*) as follows:

(1) $$OP=\frac{OTR\cdot e}{\Delta P}$$

(2) $$WVP=\frac{WVTR\cdot e}{\Delta P}$$

where ΔP is the partial-pressure difference of the permeant across the film.

#### 2.3.2. Optical Properties

The film color was quantified with a CR-200 tristimulus colorimeter (Konica Minolta Sensing Inc., Tokyo, Japan) using the CIELAB space, where L* denotes lightness and a* and b* are the chromaticity coordinates spanning green(−)/red(+) and blue(−)/yellow(+), respectively. During acquisition, the specimens were placed on the instrument’s standard white reference plate (L* = 97.34, a* = 0.06, b* = 1.82). Optical transmittance was determined at 600 nm (T_600_) with a Libra S22 spectrophotometer (Biochrom, Cambridge, UK). Rectangular strips (25 mm × 10 mm) were positioned flush against one face of the cuvette; an empty cuvette served as the blank. The film opacity was calculated as the ratio of the absorbance at 600 nm to the film thickness (mm). For each material, three independently prepared film replicates were analyzed, with three readings performed per replicate.

### 2.4. Respiration Rate of Fresh-Peeled Garlic Cloves

The respiration rate of peeled garlic cloves was determined using a respirometer adapted from the Arduino-based system described by González-Buesa and Salvador [39]. The system was designed to determine the respiration rates under closed-system conditions by continuously monitoring the O_2_ and CO_2_ concentrations in the headspace of an airtight respiration chamber. The barometric pressure, the differential pressure between the chamber interior and the external environment, and the temperature were also recorded. For each assay, 200 ± 20 g of peeled garlic cloves was placed in the respiration chamber, leaving a free headspace volume of 800 ± 20 mL. The assays were conducted at 5 °C. Data were recorded every 30 s throughout the experimental period, and four independent assays were performed.

The partial pressures of O_2_ and CO_2_ were calculated from the gas volumetric fractions measured by the respirometer sensors using the following equation:

(3) $$p_{i}=y_{i}P_{in}$$

where $p_{i}$ is the partial pressure of gas i, $y_{i}$ is the corresponding volumetric fraction in the headspace, and $P_{in}$ is the internal pressure in the respiration chamber, calculated from the external barometric pressure and the differential pressure measured between the inside and outside of the chamber.

The O_2_ consumption rate, $R_{O_{2}}$, and the CO_2_ production rate, $R_{{CO}_{2}}$, were calculated using the following equations:

(4) $$R_{O_{2}}=-\frac{V_{h}}{WRT}\;\frac{\left(p_{O_{2},\;t_{2}}-p_{O_{2},\;t_{1}}\right)}{t_{2}-t_{1}}{\;V}_{m,\;STP}$$

(5) $$R_{CO_{2}}=\frac{V_{h}}{WRT}\;\frac{\left(p_{{CO}_{2},\;t_{2}}-p_{{CO}_{2},\;t_{1}}\right)}{t_{2}-t_{1}}\;V_{m,STP}$$

where $V_{h}$ is the free headspace volume; W is the product mass; R is the ideal gas constant; T is the absolute temperature; t is the time; $p_{O_{2}}$ and $p_{{CO}_{2}}$ are the partial pressures of O_2_ and CO_2_, respectively; and $V_{m,STP}$ is the molar volume under standard temperature and pressure conditions. The respiration rates are expressed as mL·kg^−1^·h^−1^. The observed respiration rates were subsequently fitted to different empirical and enzyme kinetic models for use as input in the microperforated packaging model. The evaluated models included polynomial empirical equations; a linear model with an intercept; a simple Michaelis–Menten model; and Michaelis–Menten models incorporating competitive, uncompetitive, noncompetitive, and mixed inhibition by CO_2_. In all Michaelis–Menten-type models, respiration was described as a function of the internal O_2_ partial pressure, whereas the inhibition models additionally included the internal CO_2_ partial pressure. Model selection was based on the root mean square error (RMSE), the model parsimony, and the biological interpretability of the fitted kinetic parameters.

### 2.5. MAP of Fresh-Peeled Garlic Cloves

Approximately 150 g of fresh-peeled garlic cloves was placed in a 500 mL polypropylene tray. The upper surface of the package (87.75 cm^2^) was sealed with one of five lid films: an uncoated EWP (EWP-U) film, a beeswax-coated EWP (EWP-BW) film, a polylactic acid (PLA) film, an oriented polypropylene (OPP) film, or a macroperforated OPP film with six 5 mm diameter perforations as the control (C). All films except the control were microperforated with a needle (0.6 mm in diameter), with five microperforations performed per package. Because needle microperforation may result in variability between individual holes and film materials, as well as deviations from an ideal circular geometry due to material deformation, edge irregularities, and partial material retention around the perforation, an equivalent perforation diameter was estimated. This equivalent diameter was calculated using a mathematical model developed by González-Buesa et al. [40]; it simulates the evolution of the gas composition inside microperforated packages. Given the size and number of the perforations and the characteristics of the tray and films, the main gas exchange route through the film was assumed to occur through the microperforations. The equivalent perforation diameter was estimated by running the model with different candidate diameters ranging from 30 to 280 µm in 5 µm increments and selecting the value that minimized the joint root mean square error (RMSE) between the experimental and model-predicted O_2_ and CO_2_ evolution in the package headspace. The uncertainty was assessed using 1000 nonparametric cluster bootstrap samples, in which complete paired O_2_ and CO_2_ experimental trajectories were resampled at the package level and the optimal diameter was re-estimated for each bootstrap sample.

The initial headspace composition matched that of ambient air. The packages were stored at 5.0 ± 0.5 °C and a 66.5 ± 1.7% RH for 28 days. The headspace O_2_ and CO_2_ concentrations were measured 48 h after packaging and subsequently at 7-day intervals using a CheckMate II gas analyzer (PBI Dansensor, Ringsted, Denmark). For each sample time, the values represent the mean of three independent packages. Additionally, the relative humidity generated inside the trays was evaluated. For this purpose, high-accuracy SHT85 dual temperature and humidity sensors (Sensirion AG, Staefa, Switzerland) were placed inside the trays before they were filled with the fresh-peeled garlic and sealed with the appropriate lid. In parallel, one tray without a protective film was left as a reference to monitor the relative humidity of the storage chamber.

### 2.6. Quality Assessment of Fresh-Peeled Garlic Cloves

Physicochemical and microbiological analyses of the garlic cloves were conducted at 7-day intervals. For each packaging material and sampling time point, three independent trays were analyzed separately and considered experimental replicates (*n* = 3). The results were expressed as the mean of the three trays.

#### 2.6.1. Weight Loss

The weight loss of the garlic cloves was determined gravimetrically using a Sartorius 3716 balance (Sartorius, Göttingen, Germany). It was calculated as the difference between the initial mass and the mass at each sampling time and expressed as a percentage relative to the initial mass. For each time point, the results are reported as the mean of three independently packaged units.

#### 2.6.2. Fungal Decay and Visual Assessment

For each packaging condition and storage time, garlic cloves were extracted from three different packages and visually inspected and photographed. The decay incidence was quantified as the percentage of cloves exhibiting visible mold growth and was reported as the percentage of affected cloves in relation to the total number of garlic cloves inspected per treatment.

#### 2.6.3. Color

The color of the convex side of each garlic clove was determined using a CR-400 colorimeter (Konica Minolta Sensing Inc., Tokyo, Japan). The instrument was calibrated against the standard white reference plate, and measurements were recorded in the CIELAB color space. For each packaging condition, the color was determined for 15 garlic cloves randomly selected from three packages (five cloves per package). Two measurements were taken at different points within the central area of the convex side of each clove.

#### 2.6.4. Texture

The firmness of the garlic cloves was defined by compression and shear tests using a texture analyzer, TA-XT2i Plus (Stable Micro Systems, Godalming, UK), equipped with a 30 kg load cell. The compression test was conducted using a cylindrical probe with a 50 mm diameter (SMSP/50) at a crosshead speed of 1 mm·s^−1^ to a deformation of 30%. The shear tests were performed with a Warner–Bratzler blade acting at 1 mm·s^−1^. The firmness was taken as the maximum force recorded in each test. Measurements were performed on 15 garlic cloves, with five cloves randomly selected from each of three packages. Each clove was oriented with its longitudinal axis perpendicular to the probe’s travel direction and the most convex surface facing upward.

#### 2.6.5. Phenolic Compounds

For each condition, garlic samples were collected from three independent packages; their total phenolic content was analyzed using the spectrophotometric method (Folin–Ciocalteu) and their profile was determined using chromatography (UHPLC-MS/MS).

Approximately 3 g of peeled garlic cloves from each package was weighed, freeze-dried, and ground using a mortar. The extraction of phenolic compounds for each powdered sample was carried out in triplicate. For each replicate, approximately 100 mg of lyophilized material was weighed into an Eppendorf tube. Methanol (500 µL) was added, and the mixture was vortexed for 20 s. The samples were then sonicated in an ultrasonic bath for 20 min and centrifuged at 13,500× *g* and 4 °C for 15 min. The supernatant was collected, and the extraction was repeated twice more on the remaining pellet, resulting in three successive extraction cycles per tube. Finally, the aliquots from both extraction replicates were quantitatively pooled into a single vessel for subsequent processing.

The total phenolic content (TPC) was determined using the Folin–Ciocalteu method [41] adapted to the UV/VIS microplate spectrophotometer. The Folin–Ciocalteu reagent (80 μL), water (80 μL), and the extract (10 μL) were dispensed into each well of a 96-well plate. After 3 min, 30 μL of a 7.5% sodium carbonate aqueous solution was added, and the plate was incubated in darkness at room temperature for 1 h. The absorbance was measured at 765 nm using a UV-3100PC spectrophotometer (VWR, Leuven, Belgium). The results were calculated from a gallic acid calibration curve (0–160 mg·L^−1^) and expressed as the mg gallic acid equivalents per 100 g fresh weight (mg GAE·100·g^−1^ FW). Each extract was analyzed in triplicate.

The phenolic profile was analyzed with UHPLC-TIMS-TOF-MS/MS using a Bruker Elute UHPLC system (Bruker, Bremen, Germany). Non-targeted phenolic profiling was performed via a chromatographic method adapted from Mena et al. [42]. Chromatographic separation was achieved on an Agilent Zorbax Eclipse Plus C18 column (1.8 µm, 2.1 mm × 100 mm). The analyses were carried out in the negative electrospray ionization mode. The mobile phases consisted of water containing 0.1% formic acid (mobile phase A) and acetonitrile containing 0.1% formic acid (mobile phase B). The flow rate was set at 0.5 mL· min^−1^, and the injection volume was 7 µL. The following gradient was applied: 0.0–1.0 min, 90% A and 10% B; 1.0–10.0 min, linear gradient to 5% A and 95% B; and 10.0–20.0 min, 5% A and 95% B. The column was re-equilibrated for 4 min before the next injection. The mass spectrometer was operated using predefined “3D-Metabolomics” methods consisting of data-dependent MS/MS acquisition. Nitrogen was used as the nebulizer, dry, and collision gas. The source parameters were as follows: nebulizer gas pressure, 2.2 bar; drying gas flow, 10 L·min^−1^; dry gas temperature, 220 °C. Data were acquired over an *m*/*z* range of 20–1300 at a spectral acquisition rate of 12 Hz. The cycle time was 0.5 s, with each cycle consisting of one full-scan MS spectrum followed by 5 MS/MS spectra acquired at a collision energy of 20 eV.

The data were processed using Bruker’s Metaboscape software version 2023b (Bruker, Bremen, Germany). Chromatographic alignment and feature extraction were performed, followed by metabolite annotation. Putative annotations were assigned using several databases, including the Bruker HMDB Metabolite Library, MoNA—MassBank of North America, and MS-DIAL public MS/MS. Since the method is non-targeted and the results are qualitative, only the presence of alliin was assessed, as it produced one of the most prominent analytical signals among the compounds detected.

#### 2.6.6. Microbiological Analyses

Garlic cloves stored under different MAP systems were analyzed weekly over 28 days to enumerate aerobic mesophilic microorganisms (AMMs), aerobic psychrotrophic microorganisms (APMs), and molds and yeasts. Each sample (approximately 20 g) was serially diluted in 0.1% sterile distilled peptone water (Merck, Darmstadt, Germany) and homogenized using a Stomacher 400 Circulator (Seward, London, UK) for 2 min at 250 rpm following ISO 6887-1:2017 [43]. Serial decimal dilutions were prepared in 0.1% peptone water and plated in duplicate. The culture media and incubation conditions for each microbial group were as follows: AMMs on plate count agar (PCA) (Merck) at 30 ± 1 °C for 72 h, APMs on PCA at 7 ± 1 °C for 7 days, and molds and yeasts on dichloran rose-bengal chloramphenicol agar (DRBC) (Merck) at 25 ± 1 °C for 4 days. For each sampling day, the reported value corresponds to the mean of three independently packaged units per packaging condition and is expressed as Log CFU·g^−1^.

### 2.7. Statistical Analysis

Statistical analyses were performed using GraphPad Prism version 10 (GraphPad Software, San Diego, CA, USA). The results are reported as the mean ± standard deviation (SD). Differences between the groups were assessed using a one-way ANOVA followed by Tukey’s post hoc test or Fisher’s least significant difference (LSD) test, as appropriate. Differences were considered statistically significant at *p* < 0.05. To quantify the magnitude of the effects identified by the two-way ANOVA, the partial eta-squared $\left(\eta_{p}^{2}\right)$ was calculated as follows:

$$\eta_{p}^{2}=\frac{SS_{\mathrm{effect}}}{\left(SS_{\mathrm{effect}}+SS_{\mathrm{error}}\right)}$$

where $SS_{\mathrm{effect}}\;$ is the sum of the squares attributable to the factor under evaluation and $SS_{\mathrm{error}}$ is the residual sum of the squares. This effect-size measure ranges from 0 to 1 and was used to assess the relative importance of the packaging system, storage time, and their interaction, thereby complementing the *p*-value, which only indicates statistical significance.

## 3. Results and Discussion

### 3.1. Barrier and Optical Properties of the Films

#### 3.1.1. Oxygen and Water Vapor Permeability

**Figure 1:**
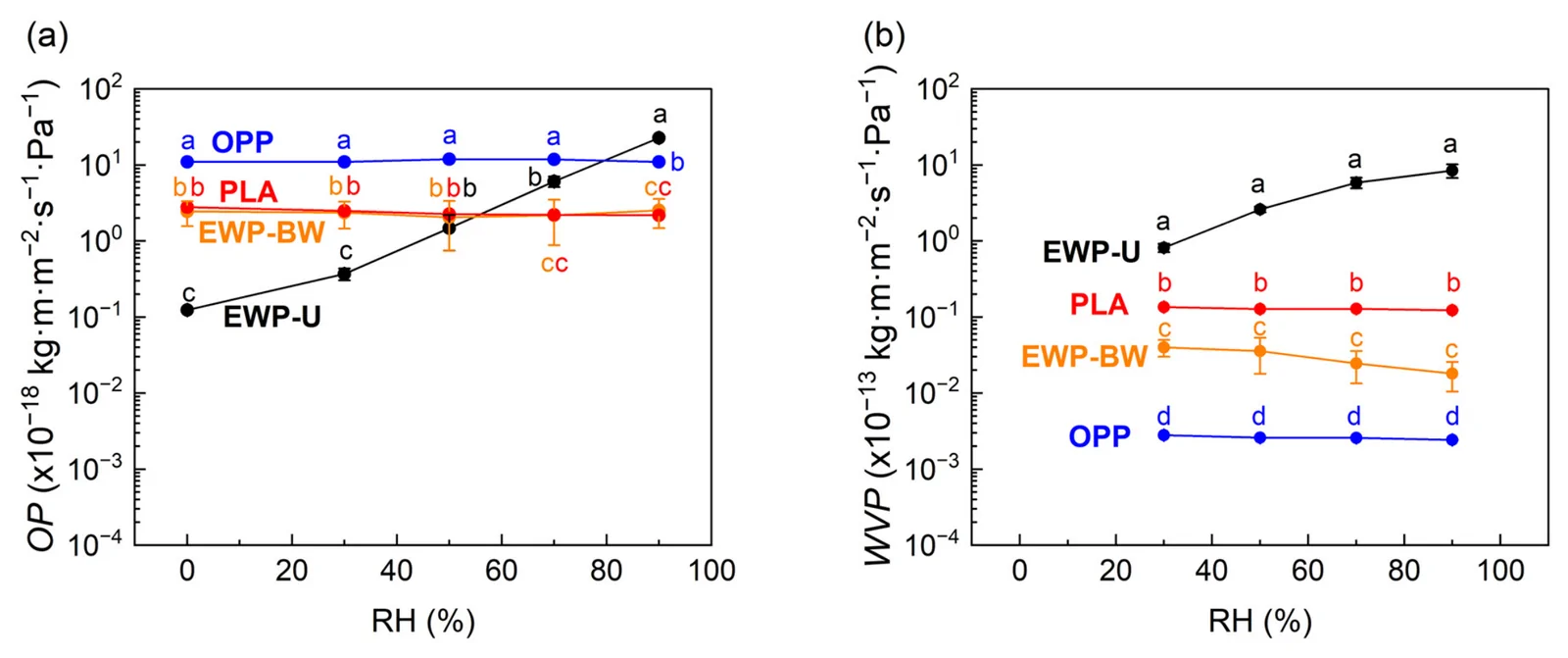
(**a**) Oxygen permeability (*OP*) and (**b**) water vapor permeability (*WVP*) of EWP-U, EWP-BW, PLA, and OPP films, measured at 23 °C under different relative humidity (RH) conditions. For each film material, six independently prepared specimens were tested. Different lowercase letters indicate significant differences between films at the same RH (*p* < 0.05).

The *OPs* of the uncoated EWP (EWP-U), beeswax-coated EWP (EWP-BW), and commercial PLA and OPP films at 23 °C are shown in Figure 1a. The *OP* of the EWP-U film was strongly dependent on the RH, increasing from 0.12 ± 0.07 × 10^−18^ to 22.76 ± 0.23 × 10^−18^ kg·m·m^−2^·s^−1^·Pa^−1^ as the RH rose from 0% to 90%. An exponential increase in the *OP* above ~60% RH has been reported previously for EWP films [14]. This behavior is commonly attributed to the hygroscopic nature of proteins: water uptake plasticizes the matrix and increases the oxygen solubility, thereby enhancing permeation. When the EWP film was coated with beeswax (EWP-BW), the *OP* became effectively insensitive to the RH, with values between 2.04 ± 1.31 × 10^−18^ and 2.53 ± 1.05 × 10^−18^ kg·m·m^−2^·s^−1^·Pa^−1^ across 0–90% RH. Thus, under low-RH conditions, the beeswax coating provides a limited additional oxygen-barrier effect compared with uncoated EWP. At a high RH, however, the hydrophobic nature of the wax layer likely restricts moisture uptake by the protein matrix, thereby attenuating the RH-induced increase in the *OP*. This interpretation is in line with previous studies showing that hydrophobic surface coatings, such as shellac on EWP films [36] or beeswax on fungal biomass [44], did not significantly affect the *OP* at ~50% RH. In contrast, in other protein-based systems such as soy protein isolate films, the incorporation of beeswax into the polymer phase has been reported to increase the *OP* [45]. This effect is attributed to the formation of interfacial discontinuities within the film structure, which may create preferential pathways for oxygen transport. The commercial PLA film exhibited an RH-independent *OP*, with values of 2.18 ± 0.01 × 10^−18^ to 2.78 ± 0.01 × 10^−18^ kg·m·m^−2^·s^−1^·Pa^−1^ over 0–90% RH. These values were similar to those obtained for the EWP-BW film, with no significant differences between the two materials (*p* > 0.05). By contrast, the commercial OPP films showed a higher *OP* than the PLA and EWP-BW films (*p* < 0.05) while remaining RH-independent, with values ranging from 11.00 ± 0.59 × 10^−18^ to 11.93 ± 0.60 × 10^−18^ kg·m·m^−2^·s^−1^·Pa^−1^ over the tested RH range.

The *WVP* of the films is presented in Figure 1b. Analogous to the *OP*, the *WVP* of the EWP-U films was highly sensitive to the RH, increasing approximately fourfold as the RH rose from 30% to 90% (from 0.81 ± 0.22 × 10^−13^ to 8.45 ± 1.70 × 10^−13^ kg·m·m^−2^·s^−1^·Pa^−1^). This trend is consistent with previous observations for EWP films produced by extrusion and calendering [14]. The hygroscopic nature of the proteins underpins similar RH-dependent behavior reported for other protein-based materials, such as soy protein films [46]. The beeswax surface-coated EWP films (EWP-BW) exhibited a reduced *WVP* by roughly two orders of magnitude and a suppressed RH dependence compared with the EWP films, yielding values of 4.0–1.8 × 10^−15^ kg·m·m^−2^·s^−1^·Pa^−1^ across 30–90% RH. The water vapor barrier properties were enhanced in some cases by the addition of a hydrophobic beeswax layer to protein-based films [44]. More commonly, beeswax is incorporated as an emulsion within the polymer matrix, which also effectively lowers the *WVP* of other protein-based films [47,48]. Similar reductions have been observed in other biopolymers using a beeswax-based multilayer strategy [25,49,50]. The *WVP* of the beeswax-coated EWP was lower than that of commercial bio-based PLA—which was also RH-independent (0.81–1.28 × 10^−14^ kg·m·m^−2^·s^−1^·Pa^−1^)—whereas commercial OPP exhibited a substantially lower *WVP* than all other films at any RH (≈3 × 10^−16^ kg·m·m^−2^·s^−1^·Pa^−1^). At each RH tested, significant differences in the *WVP* were observed between all four film types (*p* < 0.05).

#### 3.1.2. Optical and Mechanical Properties

**Figure 2:**
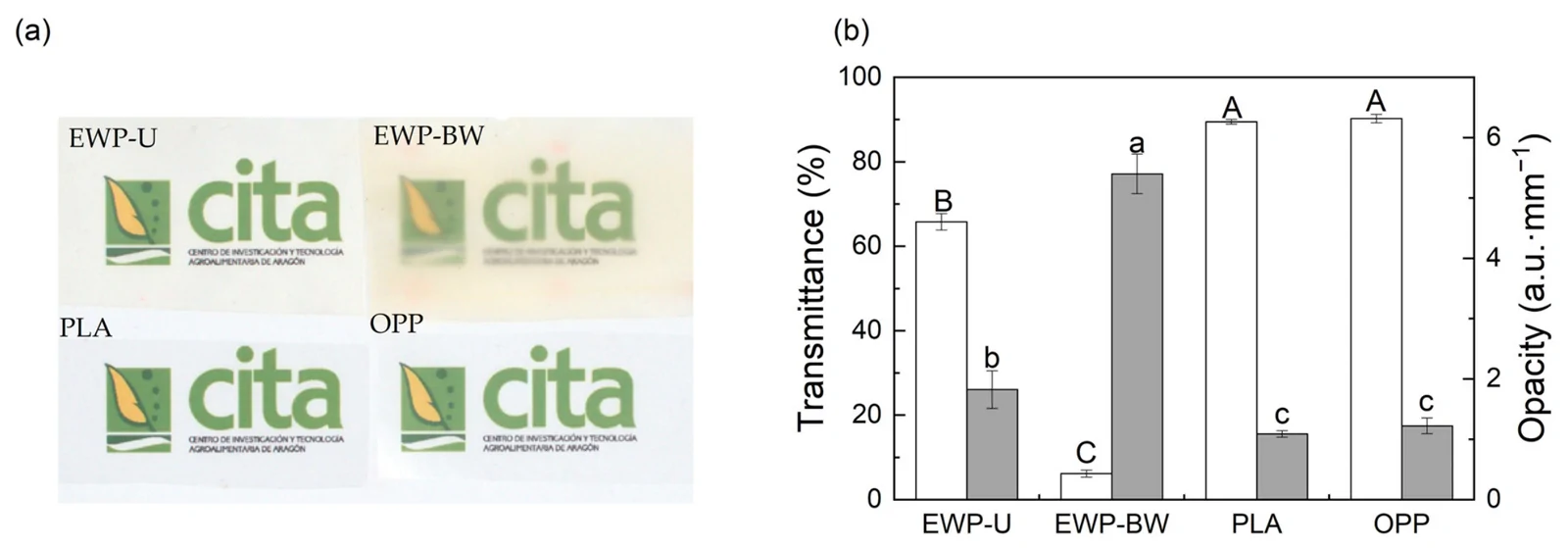
(**a**) Visual appearance placed over a color reference image (institutional logo) and (**b**) optical properties, including transmittance at 600 nm (white bars) and opacity (gray bars), of EWP-U, EWP-BW, PLA, and OPP films. Different uppercase letters indicate significant differences in transmittance between packaging systems, whereas different lowercase letters indicate significant differences in opacity between packaging systems (*p* < 0.05).

As evidenced by the photographs in Figure 2a, the EWP-U film showed a lower transparency than the commercial PLA and OPP films, with a further decrease observed after beeswax coating. The incorporation of beeswax markedly reduced the transmittance in the visible and ultraviolet regions, which can be attributed to visible-light scattering and UV absorption by beeswax crystals [51]. The transmittance at 600 nm (T_600_) and the opacity values are shown in Figure 2b. The OPP and PLA displayed T_600_ values above 80%, consistent with their classification as transparent films. By contrast, the EWP-U film fell within the translucent range, with a T_600_ value of 65.8 ± 1.9%, slightly lower than the previously reported values for EWP films [14,31]. This likely reflects the differences in the film-forming conditions and processing methods. After applying a beeswax coating, the EWP film became nearly opaque, showing a T_600_ value of only 6.2 ± 0.8%. This pronounced loss of transparency is consistent with the increased opacity reported for other bio-based films containing beeswax, where the wax phase was used to enhance the barrier performance [45,47,48,50]. Although the EWP-BW film became nearly opaque, the garlic cloves remained visible through the transparent sides and bottom of the tray; nevertheless, the reduced visibility from the top may represent a practical limitation for its commercial application.

Preliminary mechanical characterization was performed using multiaxial and puncture tests. No significant differences in maximum force were observed between coated and uncoated films. Additionally, the mechanical properties of uncoated EWP films, including tensile strength, elongation at break, and elastic modulus, were previously characterized experimentally in our earlier work [9]. Since the BW coating was applied at 70 °C, a temperature well below both the thermal melting peak (*T_m_*) of EWP and the processing temperature used for film formation (130 °C). Therefore, the coating process itself is not expected to substantially alter the mechanical properties of the EWP matrix. Consistent with this assumption, previous studies on comparable biopolymer films have shown that beeswax surface coatings do not necessarily compromise the mechanical integrity of the underlying polymer matrix [52,53]. However, the thickness of the uncoated films was approximately 120 µm, whereas application of the BW coating increased the total film thickness to approximately 260 µm. This increase should be carefully considered when interpreting the tensile response, as the measured mechanical properties may be affected by changes in sample thickness. Consequently, although the preliminary results suggest that BW coating does not affect the maximum force sustained by the films, further mechanical testing is warranted to comprehensively evaluate the mechanical performance of EWP-BW films.

### 3.2. Respiration Kinetics of Fresh-Peeled Garlic Cloves

Although the Michaelis–Menten models that included CO_2_ inhibition provided lower RMSE values in some cases, the estimated inhibition constants were high and showed considerable variability between replicates. In particular, the uncompetitive inhibition model yielded *K_iu_* values ranging from 19.4 to 65.3% for O_2_ consumption and from 16.8 to 67.2% for CO_2_ production. This suggests that the CO_2_ inhibition term was weakly identifiable under the experimental conditions tested. Therefore, the simple Michaelis–Menten model was selected for subsequent package simulations, as it offered an appropriate balance between goodness of fit, model simplicity and biological interpretability.

**Figure 3:**
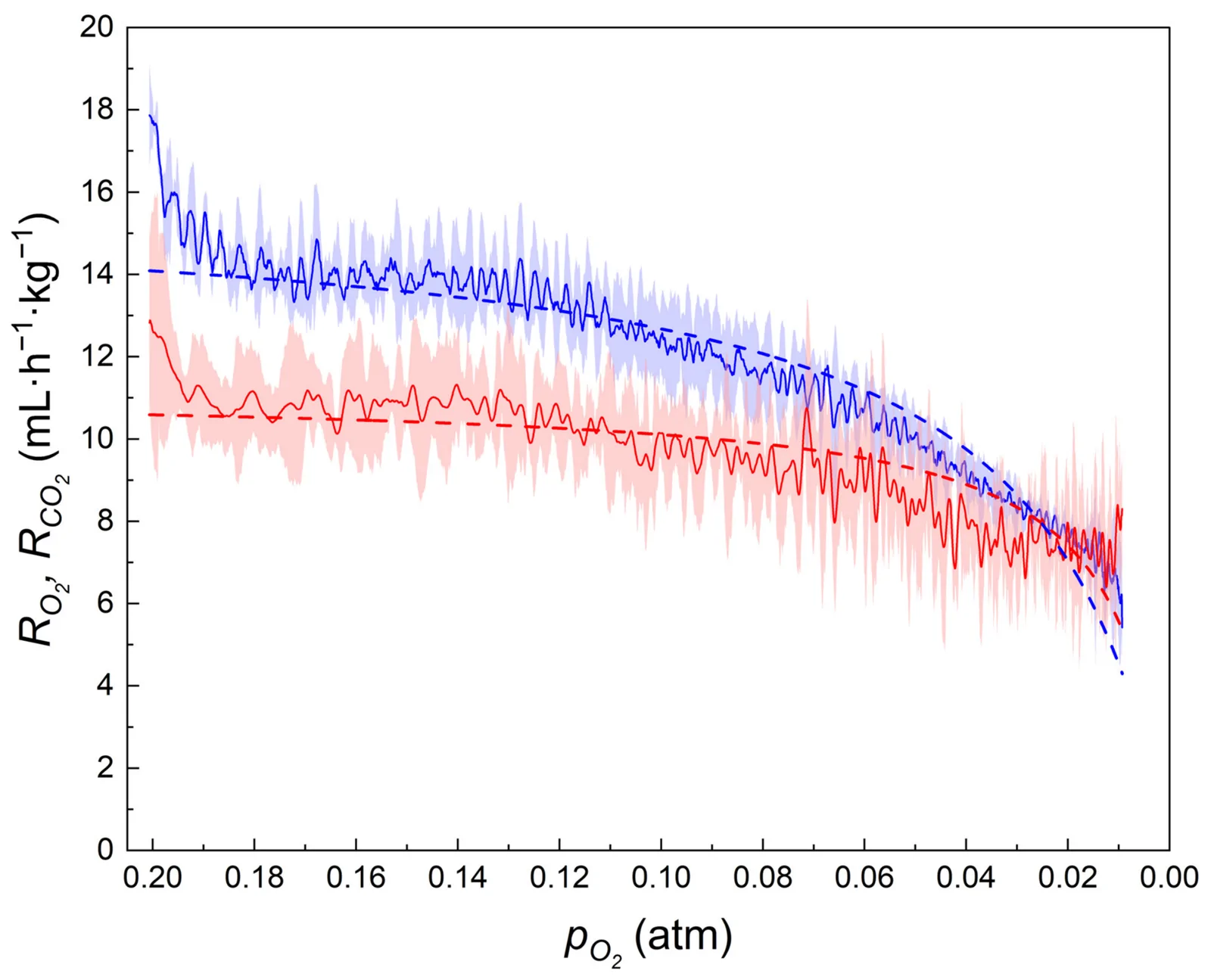
O_2_ consumption rate ($R_{O_{2}};$ blue lines) and CO_2_ production rate ($R_{{CO}_{2}};$ red lines) of peeled garlic cloves at 5 °C as a function of O_2_ partial pressure ($p_{O_{2}}$). Solid lines represent experimental data, whereas dashed lines represent data fitted to Michaelis–Menten kinetics. Blue and red shaded areas indicate the experimental standard deviation.

Figure 3 shows the O_2_ consumption and CO_2_ production rates of peeled garlic cloves at 5 °C, calculated from the experimental data using Equations (4) and (5). The resulting respiration rates were fitted to Michaelis–Menten enzyme kinetics according to the following equations:

(6) $$R_{O_{2}}=\frac{V_{max,O_{2\;}\cdot {\;p}_{O_{2}}}}{K_{m,O_{2\;}+{\;p}_{O_{2}}}\;}$$

(7) $$R_{{CO}_{2}}=\frac{V_{max,{CO}_{2}\;\cdot \;p_{O_{2}}}}{K_{m,{CO}_{2}\;+\;p_{O_{2}}}}$$

where $V_{max}$ is the maximum respiration rate and $K_{m}$ is the partial pressure at which the respiration rate reaches half of $V_{max}$. Michaelis–Menten fitting yielded $V_{max,O_{2}}$ = 15.846 ± 0.762 mL·kg^−1^·h^−1^ and $K_{m,O_{2}}$ = 0.025 ± 0.006 atm for O_2_ consumption, with an RMSE of 0.822 mL·kg^−1^·h^−1^. For CO_2_ production, the corresponding values were $V_{max,{CO}_{2}}$ = 11.118 ± 1.159 mL·kg^−1^·h^−1^ and $K_{m,{CO}_{2}}$ = 0.010 ± 0.004 atm, with an RMSE of 1.328 mL·kg^−1^·h^−1^. The fitted curves are also included in Figure 3.

Few studies have specifically evaluated the respiration behavior of peeled garlic cloves under modified atmosphere conditions. Previous studies have modeled the respiration kinetics of peeled garlic cloves using Michaelis–Menten-type equations incorporating uncompetitive inhibition by CO_2_ [5]. At 5 °C, the kinetics proposed by Lee et al. [5] predicted respiration rates in air of 15.6 and 10.2 mL·kg^−1^·h^−1^ for O_2_ consumption and CO_2_ production, respectively, which were close to the values obtained in the present study: 14.1 and 10.6 mL·kg^−1^·h^−1^, respectively. However, slightly higher respiration rates were reported by Gorrepati and Bhagat [54], who obtained a CO_2_ production rate of 16.6 mL CO_2_ mL·kg^−1^·h^−1^ at 4 °C. Under the conditions evaluated in the present study, the O_2_ consumption and CO_2_ production rates appeared to be governed primarily by O_2_ availability, with no clear dependence on the CO_2_ concentration. This lack of a significant CO_2_ effect on the respiration rate is consistent with the findings of Madhav et al. [9], who reported that varying the CO_2_ levels between 5 and 15% at a fixed O_2_ concentration did not produce significant changes in the respiration rate.

### 3.3. Evolution of In-Package Gas Composition During Storage

**Figure 4:**
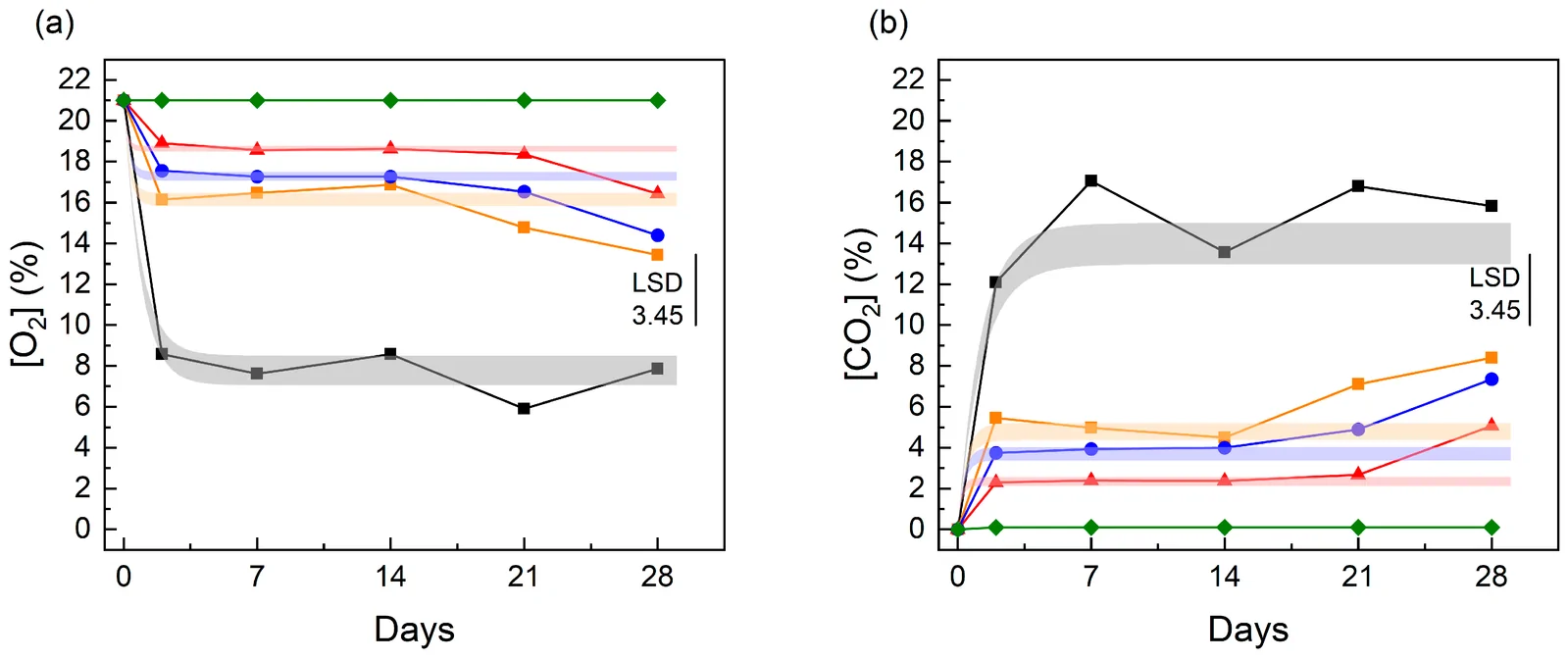
Evolution of O_2_ (**a**) and CO_2_ (**b**) concentrations inside fresh-peeled garlic packages using EWP-U (black), EWP-BW (orange), PLA (red), OPP (blue), and C (green) films during storage from days 0 to 28. The symbols represent experimental data, whereas the shaded areas denote the model-predicted ranges obtained by accounting for the standard deviations of the product’s estimated respiration kinetic parameters.

Figure 4 shows the headspace gas composition obtained for the different film types. The equivalent perforation diameters of the packages were estimated by minimizing the discrepancy between the experimentally measured and model-predicted headspace atmospheres, using the mathematical model proposed by González-Buesa et al. [40] with the joint RMSE for the O_2_ and CO_2_ concentrations as the objective function. This procedure yielded equivalent diameters of 60 µm for EWP-U, 125 µm for EWP-BW, 210 µm for PLA, and 150 µm for OPP, with joint RMSE values of 1.169, 0.361, 0.105, and 0.161 percentage points, respectively. The nonparametric cluster bootstrap analysis produced mean diameter estimates of 60.0, 125.3, 212.1, and 148.7 µm, respectively. The corresponding standard errors were 0.0, 5.7, 2.5, and 2.2 µm, with 95% confidence intervals of 60–60 µm for EWP-U, 110–135 µm for EWP-BW, 210–215 µm for PLA, and 145–150 µm for OPP.

Figure 4 shows that packages sealed with the EWP-U film exhibited distinct headspace gas evolution compared with the other packaging materials, leading to a more pronounced modification of the internal atmosphere. This behavior can be attributed to the smaller equivalent perforation diameter estimated for EWP, which restricted the gas exchange to a greater extent. Consequently, the EWP-U packages showed the largest decrease in the O_2_ concentration and the greatest accumulation of CO_2_ during storage. In contrast, the EWP-BW, PLA, and OPP packages exhibited a more moderate evolution of the internal atmosphere, whereas the macroperforated films (C) maintained O_2_ and CO_2_ concentrations close to atmospheric levels throughout storage. As expected, microperforations played an important role in the evolution of the package gas composition. In fact, the contribution of the film matrix to the effective O_2_ transmission rate was less than 5% in all cases. Because gas exchange occurred predominantly through the microperforations, the effective CO_2_ transmission rate can be assumed to be similar to that of O_2_. In contrast, if these films were used without microperforations, the CO_2_ permeability and gas selectivity of the film matrix would become essential parameters for predicting atmosphere evolution. Since the matrix is expected to be more permeable to CO_2_ than to O_2_, CO_2_ accumulation inside the package would be limited. Furthermore, a gradual increase in the perforation size may increase the gas transmission rate, although its effect on atmosphere evolution becomes less evident at larger perforation sizes, which result in steady-state atmospheres closer to air.

From day 21 onward, the O_2_ concentrations decreased more sharply, and the CO_2_ concentrations increased more markedly, coinciding with higher microbial counts. Microbial metabolism may therefore have contributed to the additional O_2_ consumption and CO_2_ production, as mold infection has been linked to increased respiration in other fresh products [55]. However, because respiration was not measured separately in spoiled cloves, the contributions of garlic tissue and microbial activity cannot be distinguished.

### 3.4. Quality of Fresh-Peeled Garlic Cloves in MAP

#### 3.4.1. Weight Loss

**Figure 5:**
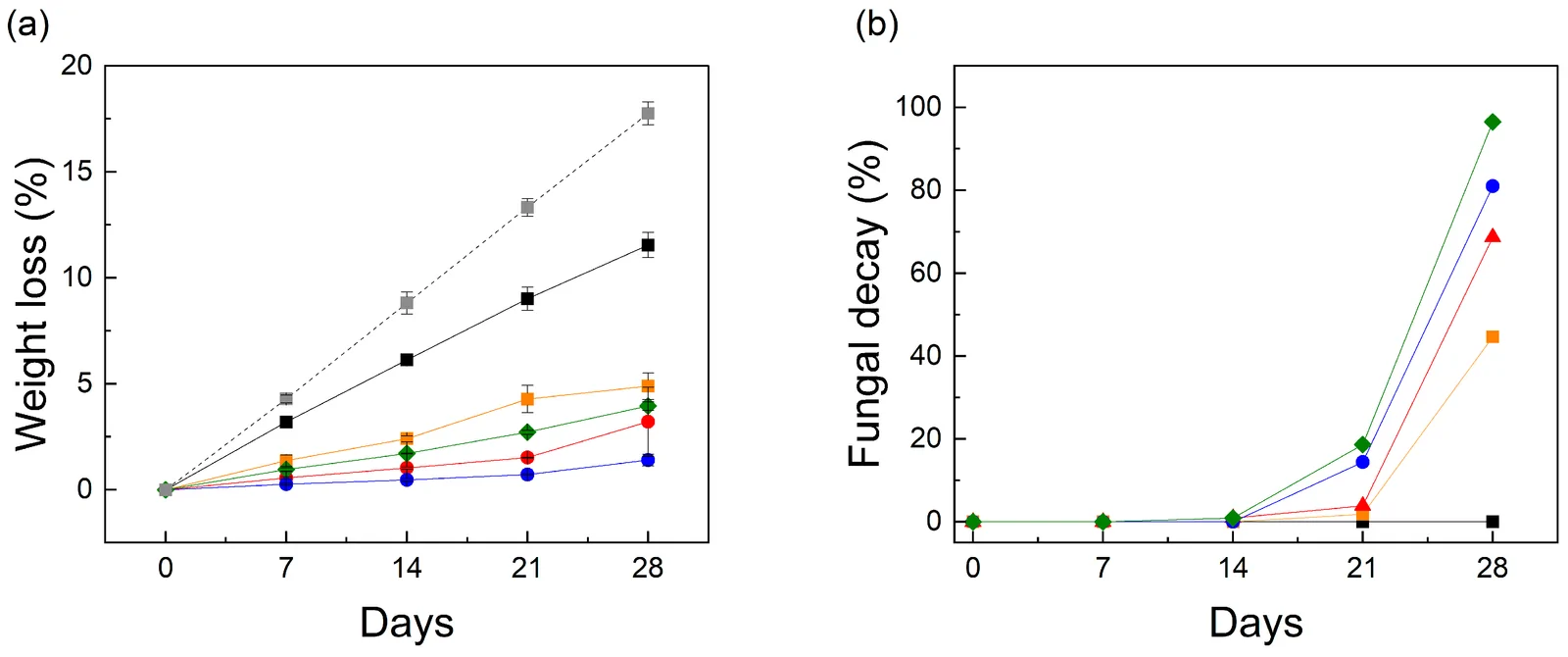
Weight loss (**a**) and incidence of fungal decay (**b**) in fresh-peeled garlic packaged using EWP-U (black), EWP-BW (orange), PLA (red), OPP (blue), and C (green) films during storage from days 0 to 28. Weight loss in samples stored in uncovered trays is also shown (gray).

Weight loss is a critical determinant of the commercial quality of fresh-peeled garlic, since excessive weight loss may promote tissue shrinkage and reduce clove firmness. Figure 5a shows the evolution of weight loss in peeled garlic under the different packaging conditions. As expected, the higher *WVP* of the EWP-U film resulted in a considerable weight loss in the garlic cloves, reaching 11.55 ± 0.60% after 28 days of storage at 5 °C. Although no universally accepted weight-loss threshold has been established for peeled garlic, this value indicates that EWP-U was less effective than the other treatments at limiting moisture loss. Therefore, this result should be considered a drawback of EWP-U during prolonged storage. At the other extreme, the weight loss of the garlic cloves stored in OPP packages was very limited (<1.5% after 28 days), as the OPP material presented a better water vapor barrier compared with the other materials. Garlic packaged in the PLA and EWP-BW films presented moderate weight loss, which is consistent with the intermediate values of *WVP* measured in these materials compared with the OPP and EWP-U films. In contrast to the gas transport for oxygen and carbon dioxide, water vapor transport through the matrix film is the main transport pathway in microperforated films, particularly in films with limited water vapor barrier properties.

Peeled garlic cloves packaged in the macroperforated film (C) showed an intermediate weight loss between that observed for the PLA and EWP-BW samples, reaching 3.95 ± 0.20% after 28 days. This result is consistent with the characteristics of the macroperforated OPP film, since the effect of the perforations on water vapor transport is more limited than for O_2_ and CO_2_, as further evidenced by the package reaching a relative humidity of 95.19 ± 2.99%. This high relative humidity could have limited the transpiration in the garlic cloves and reduced the weight loss. By contrast, samples packaged in EWP-U exhibited greater weight loss, in agreement with the higher water vapor transmission rate measured in the matrix and the lower relative humidity recorded inside the package (70.53 ± 2.36%). Exclusively for comparison purposes, the weight loss under extreme conditions was also measured by preparing additional samples that were stored in trays without any lidding film, thus matching the relative humidity (66.5 ± 1.7%) in the storage chamber. These samples exhibited the greatest weight loss, reaching 17.76 ± 0.55% by day 28.

#### 3.4.2. Fungal Decay

The conditions in the EWP-U packages limited fungal decay, since no fungal deterioration was observed in the garlic cloves over 28 days (Figure 5b). This limited fungal development was likely associated with the high CO_2_ and low O_2_ concentrations in the EWP-U package headspace, along with a reduced relative humidity around the garlic cloves. Effectively, a reduction in O_2_ in the headspace has been associated with reduced mold development in peeled garlic [1], while high relative humidity conditions inside the package may promote water condensation, favoring fungal development [56]. For the EWP-BW, PLA, and OPP packages, the relative humidity appeared to be the main factor promoting fungal deterioration, as the packages with a better water vapor barrier developed more fungal decay at the end of the storage period (while maintaining a similar O_2_ and CO_2_ composition). Garlic packaged in the macroperforated film (C) exhibited the greatest visible fungal decay, probably due to these packages having a gas composition close to the one in air along with a high relative humidity and greater cross-contamination from the storage chamber.

#### 3.4.3. Color

The evolution of the CIELAB color coordinates during storage is summarized in Table 1 for all packaging systems. Overall, storage led to a decrease in L* and an increase in a* and b*, indicating progressive darkening accompanied by a shift towards more intense red and yellow tones. These changes are consistent with enzymatic browning reactions and are mainly associated with the oxidation of garlic’s phenolic compounds in the presence of oxygen, catalyzed by polyphenol oxidase. The color changes observed in peeled garlic agree with those previously reported for garlic stored under MAP conditions [1,4,56].

No statistically significant differences between the packaging systems were detected during the first 14 days of storage (*p* > 0.05). From day 21 onward, however, samples packaged with uncoated EWP exhibited the smallest color deviations from the initial values, probably due to the lower O_2_ concentration in the package headspace. This was reflected in the total color difference (ΔE*) recorded for EWP-U on day 28 (3.26 ± 1.96), which was close to the commonly cited visual perceptibility threshold of approximately 2–3 CIELAB units. Conversely, the control packages, whose atmospheres remained close to ambient air, showed the greatest decrease in L* and the highest increase in a*, indicating more intense darkening and red color development and, consequently, the highest ΔE* value. After 28 days, the peeled garlic cloves stored in the PLA and OPP packages did not differ significantly in color (*p* > 0.05), and their CIELAB coordinates and ΔE* values were closer to those of C than to those of EWP-U.

This response may be attributed to the relatively high headspace O_2_ levels in these packages (approximately 17–19%), together with their low effective *WVP*, which limited weight loss and favored moisture retention. These conditions likely promoted enzymatic oxidation, resulting in greater darkening and yellowing than in the uncoated EWP-packaged samples. The garlic packaged in EWP-BW showed intermediate L* and a* values between those obtained with EWP-U and the commercial films, consistent with its intermediate headspace O_2_ concentration and moisture-retention behavior inferred from the weight-loss data. Overall, the reduced O_2_ availability contributed to better color preservation in fresh-peeled garlic, in agreement with previous MAP studies using oxygen scavengers [1] or atmospheres with different initial O_2_ levels [6]. However, the observed color differences may also reflect the combined effects of O_2_ availability, moisture retention, tissue integrity, the enzymatic oxidation of phenolic compounds, pH-related changes, and microbial growth.

#### 3.4.4. Texture

**Figure 6:**
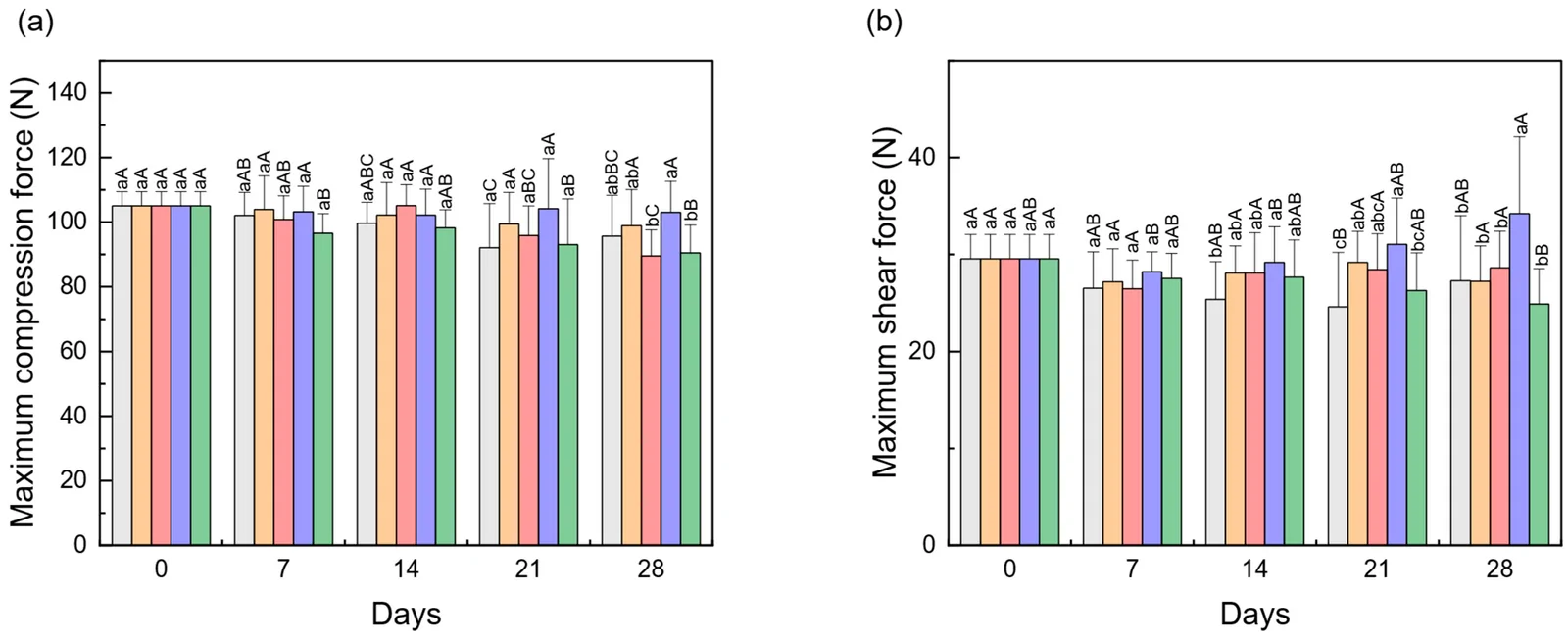
Maximum compression force (**a**) and maximum shear force (**b**) of fresh-peeled garlic packaged using EWP-U (grey), EWP-BW (orange), PLA (red), OPP (blue), and C (green) films, measured during storage from days 0 to 28. Different uppercase letters indicate significant differences between storage times within the same packaging system, whereas different lowercase letters indicate significant differences between packaging systems at the same storage time (*p* < 0.05).

The firmness of the garlic cloves, expressed as the maximum force recorded in the compression and cutting tests, is shown in Figure 6a,b, respectively, for the different packaging systems during storage. During the first 14 days, neither the compression nor the cutting force differed significantly from the initial value (*p* > 0.05). A reduction in the firmness of peeled garlic after ~14–21 days of storage has also been reported in other MAP systems [1,2,7]. Among the films tested, OPP provided the greatest retention of the initial firmness. At the end of storage, the OPP-packaged cloves were the only samples whose cutting firmness was significantly different from that of the other packaging systems.

This result is consistent with the lower weight loss observed for the OPP-packaged samples, which can be attributed to the superior water vapor barrier properties of OPP. The high RH maintained inside these packages likely reduced the moisture gradient between the garlic cloves and the package headspace, thereby limiting dehydration, the loss of cell turgor, and the reduction in firmness. Based solely on weight loss and the lower RH recorded inside the packages, cloves packaged with uncoated EWP would be expected to show the greatest firmness loss. However, this was not observed, suggesting that the modified atmosphere generated within these packages, characterized by low O_2_ and high CO_2_ concentrations, contributed to texture preservation, in agreement with the findings of Singh et al. [1]. Therefore, firmness changes appeared to be governed not only by moisture loss and the RH conditions established inside the packages but also by the headspace gas composition. This behavior may be explained by a slower progression of firmness-loss mechanisms, including dehydration-induced reductions in cell turgor and the enzymatic degradation of cell-wall polysaccharides.

#### 3.4.5. Phenolic Content and Alliin-Related UHPLC-MS/MS Analysis

**Figure 7:**
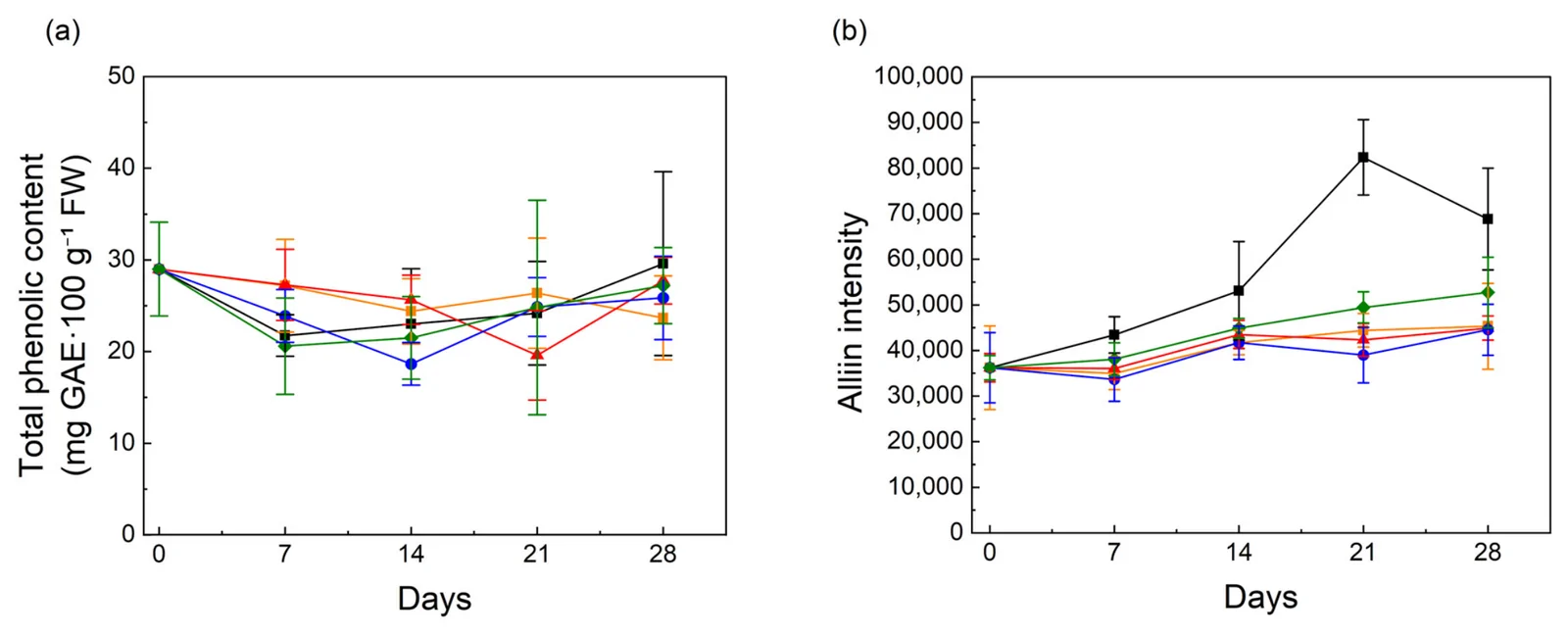
Total phenolic content (**a**) and alliin intensity (**b**) of fresh-peeled garlic packaged using EWP-U (black), EWP-BW (orange), PLA (red), OPP (blue), and C (green) films during storage from days 0 to 28.

The evolution of the total phenolic content of garlic packaged in the different films over 28 days of storage is shown in Figure 7a. At day 0, the samples presented an initial value of 29.00 ± 5.11 mg GAE·100 g^−1^ FW. After 28 days of storage, the total phenolic content showed a slight but non-significant decrease across the different packaging systems (*p* > 0.05). This was consistent with the UHPLC-MS/MS results, which showed only minor variations in the signal intensity of the detected phenolic compounds (such as gallic acid, malic acid, succinic acid, and linoleic acid). During the storage of peeled garlic, phenolic compounds may decrease as a result of polyphenol oxidase (PPO) activity, the enzyme responsible for their oxidation. In this regard, MAP with high CO_2_ and low O_2_ concentrations may help reduce enzymatic browning reactions [4]. However, under the atmospheres generated by the different packaging films used in the present study, this trend was not clearly observed, suggesting that refrigerated storage at 5 °C may be sufficient to limit phenolic compound degradation [57].

Pungency is a key quality attribute of garlic and is mainly associated with the enzymatic pathway initiated when garlic tissue is disrupted, whereby alliinase catalyzes the conversion of alliin and related S-L-cysteine sulfoxides into allicin and other sulfur-derived compounds, with pyruvic acid being formed as a reaction product. Therefore, as alliin is the main precursor involved in this pathway, changes in its levels may provide relevant information on the potential pungency and flavor development of peeled garlic [2]. The results of the UHPLC-MS/MS analysis showed differences in the intensity of the alliin peak between garlic samples stored under the different packaging systems tested (Figure 7b). Garlic samples packaged in EWP-U showed a higher alliin peak intensity than the other samples, particularly after 21 and 28 days of storage. This could be attributed to the lower O_2_ and higher CO_2_ concentrations generated in this packaging system compared with the other packaging atmospheres, which may have contributed to slowing down the enzymatic conversion of alliin. This interpretation is consistent with previous studies reporting that modified atmospheres with reduced O_2_ and increased CO_2_ can help preserve the pungency-related compounds in peeled garlic, resulting in reduced losses of pyruvic acid during storage [1,4,6,56,58].

#### 3.4.6. Effect of Packaging on Microbial Counts of Peeled Garlic

**Figure 8:**
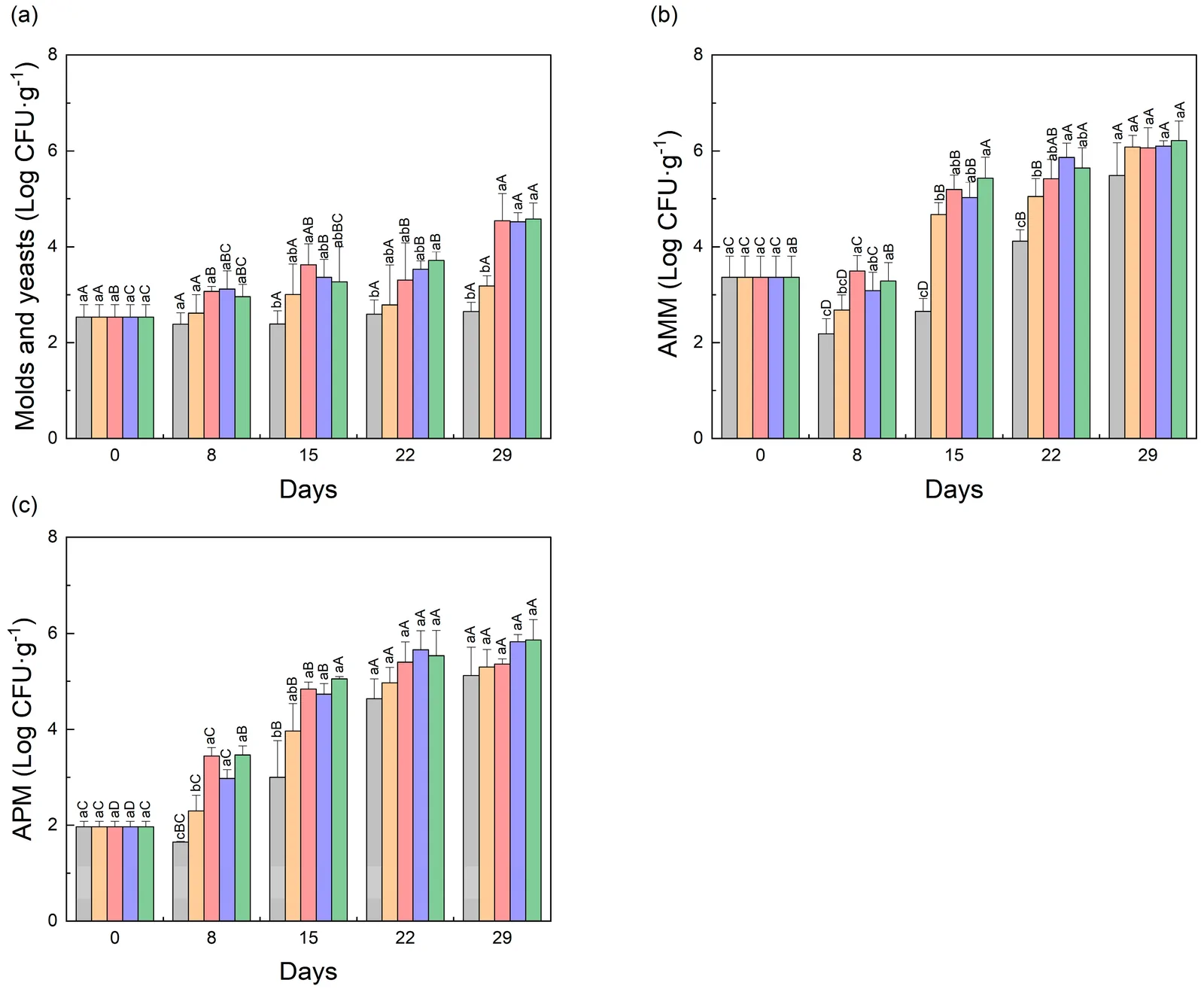
Yeast and mold counts (**a**), total aerobic mesophilic counts (AMMs) (**b**), and total aerobic psychrotrophic counts (APMs) (**c**) in fresh-peeled garlic packaged in EWP-U (gray), EWP-BW (orange), PLA (red), OPP (blue), and C (green) films, measured during storage from days 0 to 28. Different uppercase letters indicate significant differences between storage times within the same packaging system, whereas different lowercase letters indicate significant differences between packaging systems at the same storage time (*p* < 0.05).

Figure 8a reports the yeast and mold counts in peeled garlic stored under the different packaging systems. The initial fungal load was 2.5 Log CFU·g^−1^ and remained statistically unchanged in the EWP-U and EWP-BW packages throughout storage (*p* > 0.05). In contrast, the counts increased by ~2 Log CFU·g^−1^ in the samples packaged in commercial films (PLA and OPP) and in the macroperforated control (C). This behavior is consistent with the headspace conditions generated in the EWP-based packages, particularly in EWP-U, which showed the greatest CO_2_ accumulation and O_2_ depletion. These modified-atmosphere conditions likely contributed to limiting fungal growth in the EWP-U and EWP-BW packages. In addition, EWP-U maintained the lowest RH inside the package, which may have further restricted fungal development and is consistent with the lowest incidence of visible fungal spoilage (Figure 5b). Conversely, the PLA, OPP, and control packages maintained lower inhibitory gas compositions and relatively high internal RH conditions, which may have favored an increase in the yeast and mold counts. Low O_2_ and elevated CO_2_ concentrations are known to inhibit fungal growth in MAP systems and have previously been associated with reduced mold development in peeled garlic [1,56].

However, the CO_2_-rich atmosphere was insufficient to prevent the increase in the total aerobic mesophilic and psychrotrophic counts throughout storage (Figure 8b,c, respectively). The initial total aerobic mesophilic count (AMMs) was 3.4 Log CFU·g^−1^, in agreement with previous values reported for fresh-peeled garlic (~4 Log CFU·g^−1^ [1]). In EWP-based packages, the AMMs increased more slowly during the first 15 days, but subsequently accelerated, resulting in comparable final counts across all films by day 29 (5.5–6.1 Log CFU·g^−1^). The total aerobic psychrotrophic counts (APMs) followed a similar pattern, with an initial load of 2.0 Log CFU·g^−1^, slower growth up to day 15 in uncoated EWP-based packages, and final counts ranging from 5.1 to 5.9 Log CFU·g^−1^ across all films. Overall, the results indicate that the package atmosphere and the internal RH influenced fungal and bacterial populations differently: a low-O_2_/high-CO_2_ atmosphere was particularly effective at controlling fungal growth, whereas its inhibitory effect on aerobic bacterial populations was primarily transient.

Additional safety-relevant microbiological groups should be analyzed to provide a more complete microbiological assessment.

#### 3.4.7. Effect Sizes of Packaging and Storage Time on Garlic Quality Attributes

**Figure 9:**
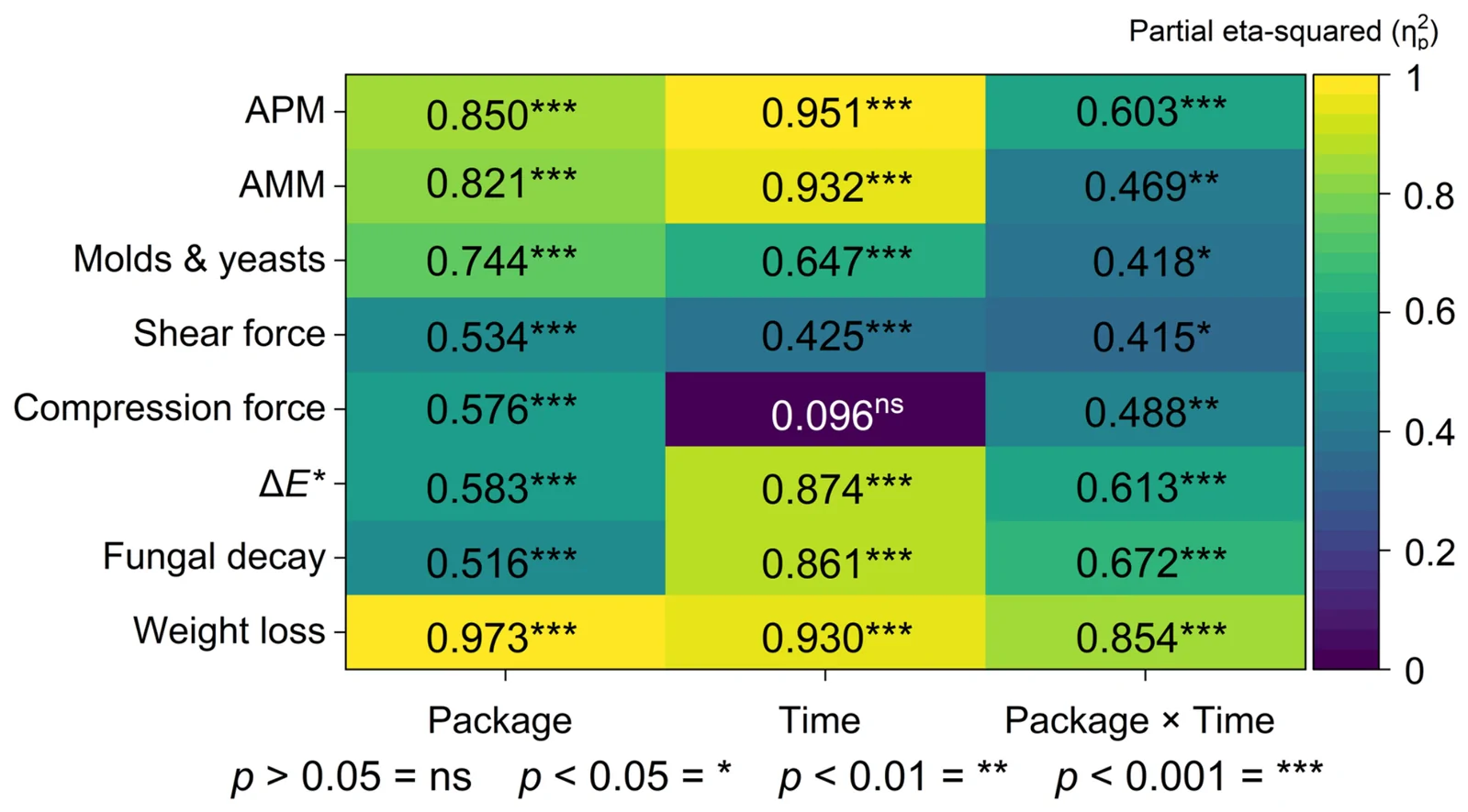
Heatmap of partial eta-squared $\left(\eta_{p}^{2}\right)$ values obtained from the two-way ANOVA, showing the effect sizes of the packaging system, the storage time, and their interaction on garlic quality attributes.

The heatmap shown in Figure 9 displays the $\eta_{p}^{2}$ values and their significance for the garlic quality parameters. Different types of packaging materials had a significant and generally large effect on all variables, with the strongest effect observed for weight loss $\left(\eta_{p}^{2}=0.973\right)$. The storage time also showed large effects on most parameters, particularly APMs $\left(\eta_{p}^{2}=0.951\right)$, AMMs $\left(\eta_{p}^{2}=0.932\right)$, and weight loss $\left(\eta_{p}^{2}=0.930\right)$, whereas its effect on compression was not significant $\left(\eta_{p}^{2}=0.096,\;p\geq 0.05\right)$. Significant packaging × time interactions were also observed for all variables, indicating that their evolution during storage depended on the packaging system, with the strongest interaction found for weight loss $\left(\eta_{p}^{2}=0.854,\;p<0.001\right)$.

## 4. Conclusions

The results obtained in this study indicate the feasibility of preserving the quality of peeled garlic cloves using bio-based microperforated MAP systems. Despite the low moisture barrier of EWP films, the establishment of an O_2_-reduced and CO_2_-enriched atmosphere inside the package contributed to maintaining several quality-related parameters of peeled garlic at levels comparable with those achieved with commercial packaging systems under air-like atmospheres. Nevertheless, the weight loss in garlic cloves packaged with uncoated EWP films was higher than in those packaged with the other materials, representing a commercially relevant drawback and highlighting the need to improve the moisture barrier properties of these protein-based films. This issue was substantially mitigated by applying a beeswax coating to both sides of the EWP film, which reduced the water vapor permeability and consequently limited product weight loss. Overall, the results indicate that improving the moisture barrier of EWP films alone is not sufficient; the microperforation pattern must also be optimized to generate a modified atmosphere with O_2_ and CO_2_ concentrations suitable for preserving the quality of peeled garlic cloves. Accordingly, properly designed microperforated EWP-BW packages represent a promising bio-based alternative to petroleum-derived packaging for the storage of ready-to-cook peeled garlic cloves, provided that the internal atmosphere generated is capable of delaying fungal decay, reducing yeast and mold growth, and alleviating surface discoloration. However, their industrial implementation will require further investigation. In particular, future studies should assess the cost and scalability of film production and the beeswax coating, the reproducibility of the manufacturing and microperforation processes on an industrial scale, the heat-sealing performance of the films, and the compatibility of the films with existing commercial packaging equipment and processing speeds. These aspects will determine the technical and economic feasibility of transferring the proposed packaging system to industrial applications.

## Figures and Tables

**Table 1:** CIELAB coordinates (L*, a*, and b*) of fresh-peeled garlic packaged using EWP-U, EWP-BW, PLA, OPP, and C films during storage from days 0 to 28. Different uppercase letters indicate significant differences between storage times within the same packaging system, whereas different lowercase letters indicate significant differences between packaging systems at the same storage time (*p* < 0.05).

	Films	0	7	14	21	28
L*	EWP-U	82.44 ± 1.46 ^aA^	80.61 ± 1.31 ^aB^	80.44 ± 2.04 ^aB^	80.13 ± 1.23 ^abB^	80.27 ± 2.05 ^aB^
	EWP-BW	82.44 ± 1.46 ^aA^	80.99 ± 1.11 ^aB^	80.44 ± 1.58 ^aB^	80.83 ± 1.45 ^aB^	78.87 ± 3.05 ^bC^
	PLA	82.44 ± 1.46 ^aA^	80.43 ± 1.32 ^aBC^	81.54 ± 0.93 ^aAB^	79.59 ± 3.10 ^abC^	77.07 ± 2.37 ^cD^
	OPP	82.44 ± 1.46 ^aA^	80.52 ± 1.07 ^aB^	80.46 ± 2.23 ^aB^	79.16 ± 2.92 ^bB^	76.93 ± 3.15 ^cC^
	C	82.44 ± 1.46 ^aA^	80.89 ± 1.50 ^aB^	80.25 ± 2.10 ^aB^	79.72 ± 1.59 ^abB^	75.13 ± 2.86 ^dC^
a*	EWP-U	1.29 ± 0.51 ^aC^	1.52 ± 0.28 ^aBC^	1.61 ± 0.31 ^bAB^	1.28 ± 0.35 ^bC^	1.84 ± 0.38 ^cA^
	EWP-BW	1.29 ± 0.51 ^aB^	1.48 ± 0.32 ^aB^	1.51 ± 0.44 ^bB^	1.68 ± 0.43 ^bB^	3.03 ± 1.55 ^bA^
	PLA	1.29 ± 0.51 ^aC^	1.66 ± 0.39 ^aC^	1.70 ± 0.38 ^abC^	2.63 ± 0.71 ^aB^	4.36 ± 1.22 ^aA^
	OPP	1.29 ± 0.51 ^aC^	1.66 ± 0.38 ^aC^	1.62 ± 0.51 ^bC^	2.70 ± 0.84 ^aB^	4.33 ± 1.32 ^aA^
	C	1.29 ± 0.51 ^aD^	1.59 ± 0.33 ^aCD^	1.94 ± 0.51 ^aC^	2.95 ± 0.76 ^aB^	5.11 ± 1.18 ^aA^
b*	EWP-U	16.86 ± 1.13 ^aC^	16.40 ± 1.05 ^bC^	17.77 ± 1.28 ^bAB^	17.21 ± 1.16 ^cBC^	18.60 ± 1.48 ^cA^
	EWP-BW	16.86 ± 1.13 ^aC^	16.89 ± 0.72 ^abC^	18.54 ± 1.33 ^abB^	19.37 ± 1.24 ^bB^	20.54 ± 2.13 ^abA^
	PLA	16.86 ± 1.13 ^aC^	17.19 ± 1.15 ^aBC^	18.22 ± 1.40 ^abB^	19.88 ± 1.57 ^abA^	20.04 ± 2.80 ^abcA^
	OPP	16.86 ± 1.13 ^aD^	17.38 ± 0.77 ^aD^	18.97 ± 1.34 ^aC^	20.40 ± 1.78 ^abB^	21.44 ± 1.77 ^aA^
	C	16.86 ± 1.13 ^aC^	16.89 ± 1.17 ^abC^	18.46 ± 1.14 ^abB^	20.54 ± 2.01 ^aA^	19.77 ± 2.54 ^bcA^
ΔE*	EWP-U		2.26 ± 1.16 ^aB^	2.93 ± 1.46 ^abAB^	2.64 ± 1.18 ^cAB^	3.26 ± 1.96 ^cA^
	EWP-BW		1.79 ± 0.87 ^aC^	2.91 ± 1.72 ^abBC^	3.42 ± 1.44 ^bcB^	6.05 ± 2.95 ^bA^
	PLA		2.49 ± 1.11 ^aC^	2.13 ± 1.11 ^bC^	4.81 ± 2.89 ^abB^	7.44 ± 2.78 ^abA^
	OPP		2.18 ± 1.08 ^aC^	3.30 ± 2.12 ^aC^	5.35 ± 2.99 ^aB^	8.14 ± 2.96 ^aA^
	C		2.24 ± 1.04 ^aC^	3.23 ± 1.79 ^abC^	5.13 ± 2.11 ^aB^	9.23 ± 2.61 ^aA^

## Data Availability

The original data presented in this study are openly available in Zenodo at http://doi.org/10.5281/zenodo.20763720.
